# Barriers to access cancer prevention services for the homeless population in four European countries

**DOI:** 10.1093/eurpub/ckac129.069

**Published:** 2022-10-25

**Authors:** T Schiffler, C Carmichael, A Gil-Salmeron, M Kouvari, P Karnaki, I Grabovac

**Affiliations:** 1 Center for Public Health, Department of Social and Preventive Medicine, Medical University of Vienna, Vienna, Austria; 2 Centre for Health, Performance and Wellbeing, Anglia Ruskin University, Cambridge, UK; 3 International Foundation for Integrated Care, Oxford, UK; 4 PROLEPSIS Institute, Athens, Greece

## Abstract

**Background:**

People experiencing homelessness (PEH) are known to have increased burden of cancer and higher cancer-related mortality when compared to the general population. These outcomes are linked to a variety of etiological factors, as well as the existence of significant barriers in accessing cancer prevention services. The aim of this study was to better understand current practices and knowledge relating to cancer prevention among PEH, health professionals and social care workers.

**Methods:**

During autumn 2021, a cross-national qualitative study was conducted within the framework of the Horizon 2020 funded CANCERLESS project. Data were collected in Austria, Greece, Spain and the UK through semi-structured interviews. Interviews were transcribed verbatim and thematically analyzed in accordance with the approach set out by Saldaña (2021).

**Results:**

In total, 69 interviews were conducted with a sample comprising 15 professionals working in homelessness support services, 19 health professionals, and 35 PEH. Two overarching themes relating to the research question were identified, namely (a) experiences and understanding of cancer prevention and treatment, and (b) considerations for program intervention. While cancer was a major source of concern, tailored cancer prevention programs for the homeless population were described as effectively non-existent, and very few homeless participants recalled being invited to a screening appointment. Health professionals also indicated that because of barriers to health care, opportunities for the early diagnosis of cancer among PEH were often being missed.

**Conclusions:**

The results indicate that PEH have limited knowledge around the importance of cancer prevention programs, and that more focused input on the part of health and social care services is required in this area. Culturally sensitive and person-centered approaches should be adopted to facilitate access to cancer prevention for PEH.

**Key messages:**

• Specialized cancer prevention and health care pathways that take account of the living conditions and support needs of PEH should be established to improve health and cancer-related outcomes.

• Cancer prevention programs should focus on improving health literacy by using accessible and tailored approaches, both for PEH and those that work directly with the homeless population.

